# An empirical tool for estimating the share of unmet need due to healthcare inefficiencies, suboptimal access, and lack of effective technologies

**DOI:** 10.1186/s12913-019-3914-7

**Published:** 2019-02-11

**Authors:** Devin Incerti, John Browne, Caroline Huber, Christine L. Baker, Geoff Makinson, Amir Goren, Richard Willke, Warren Stevens

**Affiliations:** 1 Precision Health Economics, 11100 Santa Monica Blvd #500, Los Angeles, CA 90025 USA; 2 University College Cork, College Rd, University College, Cork, Ireland; 3 Pfizer, Inc., 235 E 42nd St, New York, NY 10017 USA; 4 Kantar Health, 11 Madison Ave # 12, New York, NY 10010 USA; 5 International Society for Pharmacoeconomics and Outcomes Research, 505 Lawrence Square Blvd, South Lawrenceville, NJ 08648 USA; 6 Parexel International, 2520 Meridian Parkway, Durham, NC 27713 USA

**Keywords:** Unmet need, Quality of life, Healthcare inefficiency, Access to healthcare, Health economics, Inequality

## Abstract

**Background:**

Although there has been growing attention to the measurement of unmet need, which is the overall epidemiological burden of disease, current measures ignore the burden that could be eliminated from technological advances or more effective use of current technologies.

**Methods:**

We developed a conceptual framework and empirical tool that separates unmet need from met need and subcategorizes the causes of unmet need into suboptimal access to and ineffective use of current technologies and lack of current technologies. Statistical models were used to model the relationship between health-related quality of life (HR-QOL) and treatment utilization using data from the National Health and Wellness Survey (NHWS). Predicted HR-QOL was combined with prevalence data from the Global Burden of Disease Study (GBD) to estimate met need and the causes of unmet need due to morbidity in the US and EU5 for five diseases: rheumatoid arthritis, breast cancer, Parkinson’s disease, hepatitis C, and chronic obstructive pulmonary disease (COPD).

**Results:**

HR-QOL was positively correlated with adherence to medication and patient-perceived quality and negatively correlated with financial barriers. Met need was substantial across all disease and regions, although significant unmet need remains. While the majority of unmet need was driven by lack of technologies rather than ineffective use of current technologies, there was considerable variation across diseases and regions. Overall unmet need was largest for COPD, which had the highest prevalence of all diseases in this study.

**Conclusion:**

We developed a methodology that can inform decisions about which diseases to invest in and whether those investments should focus on improving access to currently available technologies or inventing new technologies.

**Electronic supplementary material:**

The online version of this article (10.1186/s12913-019-3914-7) contains supplementary material, which is available to authorized users.

## Background

With the rapid development of innovative health technologies and therapies, and constraints on public resources, there is a growing interest in identifying conditions with the most significant disease burden and needs to better allocate resources and improve patient health. One approach to identifying such conditions uses the measurement of unmet need, which is the overall epidemiological burden of disease [1]. Unmet need for healthcare can refer to the need for new technologies, the need for improved access to current technologies, or the imperfect use of current technologies. The overall magnitude of unmet need is driven by each of these subcategories, as well as the underlying intrinsic burden of the condition in question. Therefore, quantifying these subcategories of unmet need is critical to informing the allocation of scarce healthcare resources and prioritizing future research and development (R&D) investments by both the public and private sectors [2, 3].

Despite the interest in measuring unmet need, current measures are rudimentary and receive little attention in the health economics literature and policy discussions. A number of studies have described conceptual models of unmet need [1, 4, 5]. In these studies, measures of unmet need generally describe the gap between disease burden and the degree to which that burden can currently be overcome, but these measures ignore the burden that could be eliminated through technological advances [6–9]. Many epidemiological studies have attempted to define and estimate unmet need within specific diseases or therapeutic approaches [4, 10–15]. However, few studies have attempted to measure unmet need across multiple diseases while using uniform methodologies and outcome measures [10, 16, 17].

A further limitation of current measures of unmet need is the use of the disability-adjusted life year (DALY), which is the most popular measure of overall disease burden. DALYs are useful for comparing the aggregate burden across conditions and across countries. They are limited, however, when valuing the extent to which new technologies can reduce unmet need, because they do not express need in the same currency as cost-effectiveness, which currently is the quality-adjusted life year (QALY) [10]. Furthermore, DALYs usually are not measured at the individual patient level, which is required to link determinants of unmet need, such as insurance coverage, with treatment utilization.

In light of these limitations, and a need to better understand the needs of patients, we developed a conceptual framework and empirical tool that not only allows unmet need to be measured using a single outcome measure shared across all diseases, but also separates unmet from met need and subcategorizes the causes of unmet need.

By standardizing unmet need across diseases and stratifying unmet need by category, iterations of this framework and tool can be useful for decision-makers as they make resource allocation decisions. For example, they could be used by health technology manufacturers to prioritize R&D investments or policy-makers to inform disease areas that require stronger health system infrastructure. Through this manuscript, we aim to clearly articulate our approach to inform additional research in measuring unmet need.

## Methods

**Fig. 1:**
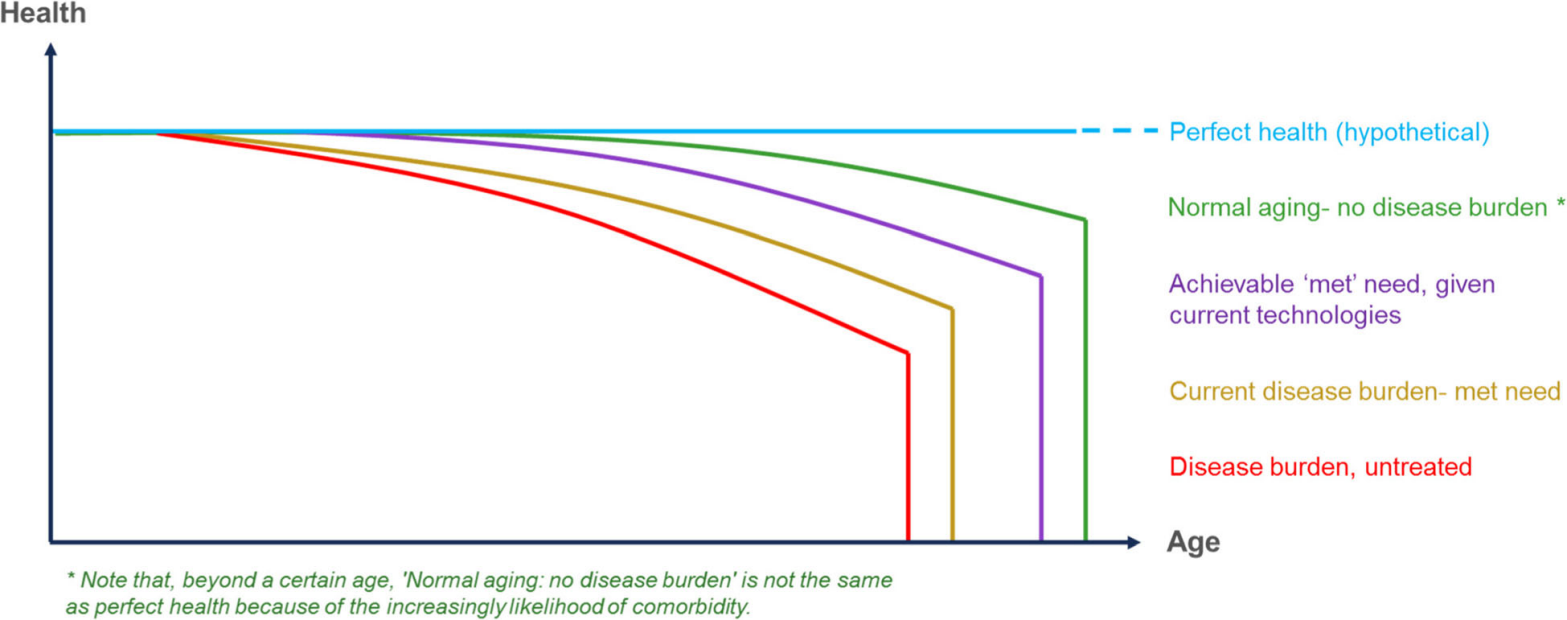
Subcategories of unmet need. Notes: This figure represents the conceptual approach to disaggregating unmet need for whole populations with a specific condition. The x-axis represents age, and the y-axis represents the disease burden of a specific condition. The approach assumes that, ceteris paribus, disease burden increases with age

Figure 1 illustrates our conceptual approach to disaggregating unmet need for whole populations with a specific condition. The figure maps health against age, and assumes that disease burden, ceteris paribus*,* increases as people age, regardless of the source of unmet need. It also assumes that, beyond a certain age, comorbidities prevent perfect health from being achieved, even if a specific condition has been eliminated through technological advances.

Our approach facilitates discrimination between sub-populations with different hypothetical levels of need. These are, in order of severity: (1) patients with untreated disease (*disease burden, untreated*); (2) patients with suboptimal access to or ineffective use of state-of-the-art technologies (*current disease burden*); (3) patients with access to and use of state-of-the-art technologies (*achievable met need*); and (4) patients receiving new technologies that eliminate the condition in question (*no disease burden*). An achieved state of (2) vs. (1) represents current *met need,* or the extent that current technologies have reduced disease burden. The gap between (4) and (2) represents remaining unmet need, which is decomposed into unmet need due to ineffective use of current technologies (gap between (3) and (2)) and lack of technologies (gap between (4) and (3)), respectively.

In principle, categories of disease burden for a given disease can be estimated using a modified version of the DALY,

1 $$ DALY\left(a,g,c\right)= YLL\left(a,g,c\right)+ YLD\left(a,g,c\right) $$

where, for a given age group *a*, gender *g*, and category *c*, YLL is years of life lost due to premature mortality and YLD is years lost due to disability. To estimate this quantity empirically, data on treatment access and use must be combined with data on morbidity and mortality. For results to be generalizable, these data must be available across a number of diseases and countries. Morbidity in the DALY framework is estimated using disability weights that are derived from a population survey; our framework replaces this with a disutility weight, equivalent to the loss of utility arising from unmet need. While we treat mortality in the same way as in the DALY framework, it is also possible to assume equivalence across conditions and use morbidity data alone when comparing chronic conditions with negligible survival impact.

### Data sources

Quantifying unmet need first requires an assessment of current disease prevalence and mortality and the level of health achievable in the various benchmark scenarios described above. The most comprehensive global source for the prevalence and mortality data required is the Global Burden of Disease Study (GBD), which provides estimates of the incidence, prevalence, and mortality of over 310 diseases and sequelae for each country, by year [18].

Second, it also requires patient-level measures of health status that can be analyzed with the main causes of unmet need. That is, the extent to which better use and access to healthcare can reduce the burden of disease must be measured, in addition to the impact of disease. Routinely-collected surveys such as the Medical Expenditure Panel Survey (MEPS) [19], the Health and Retirement Study (HRS) [20], and the National Health and Wellness Survey (NHWS) [21] provide these data. In this study, we selected the NHWS because it uses the same survey tools in multiple countries, which facilitates comparison across territories and health systems. Although a limitation of the NHWS is that it does not contain data on mortality, we determined that its multi-country coverage and rich quality-of-life data provided significant advantages over other potential data sources.

We selected five epidemiologically-diverse conditions that significantly impact morbidity, which allowed us to assess the usefulness of our empirical tool: rheumatoid arthritis (RA), breast cancer, Parkinson’s disease, hepatitis C, and chronic obstructive pulmonary disease (COPD). For each disease, we calculated years of life lost to disutility (YLD),

2 $$ YLD\left(a,g,c\right)= DW\left(a,g,c\right)\cdot p\left(a,g,c\right) $$

where *DW* is a disutility weight and *p* is the prevalence of disease. The key difference between this measure and the GBD’s “years of life lost to disability” weight is the replacement of the disability weight with the health-related quality of life (HR-QOL)-based disutility weight.

### Variables

The YLD measure was estimated separately for the United States (US) and five European countries (United Kingdom, Germany, France, Italy, and Spain, hereafter referred to as the EU5). We obtained data on prevalence by age, gender, and country from the GBD Results Tool [22].

The disutility weights were estimated using 2013 data from the NHWS. 75,000 individuals were surveyed in the US, and 62,000 individuals were surveyed in the EU5. The survey uses recent census data in each geographical region to design a sampling plan so that the survey is representative of the adult population. The survey is self-reported, cross-sectional, and online-based, but uses off-line recruitment techniques to ensure that the elderly population is well-represented.

Surveyed individuals completed the Short-Form 36-item (version 2; SF-36v2) health survey, and responses were converted into Short-Form Six-Dimension (SF-6D) utility scores using the algorithm described in Brazier et al. [23]. The utility scores range from scores below 0 representing health states worse than death to a maximum score of 1 equivalent to perfect health. Disutility was calculated as “1 – utility.” Additional details are provided in the Additional file 1.

In the NHWS, medical conditions are self-reported and based on responses to the survey questionnaire. Indicator variables were used to indicate whether a respondent had one of the five conditions of interest. We also calculated the number of comorbidities for each respondent, which were classified into four groups: zero comorbidities, one comorbidity, two commodities, or at least three comorbidities.

We measured the impact of poor access to and utilization of care by comparing utility with observed treatments to expected utility given currently available technologies. Three types of variables (four in total) were used to measure access to and use of technologies. First, the Morisky Medication Adherence Scale, 8-item (©MMAS-8) was used to measure the degree to which patients are adhering to medications. Respondents were coded according to whether they had high adherence (1) or low or medium adherence (0). Second, two variables were used as proxies for access to care. The first variable assessed whether respondents reported that costs prevented them from taking medications (1 = yes, 0 = no), and the second assessed whether respondents used a cost-cutting strategy for their medications (1 = yes, 0 = no). Third, we used a variable asking whether respondents feel their doctors are attentive to their needs and concerns (1 = yes, 0 = no), as a proxy for the quality of received care.

### Modeling disutility weights

We estimated disutility weights for different categories of YLD by modeling the relationship between treatment utilization and HR-QOL-based utility. Specifically, we used linear regression to model utility (i.e., 1 – disutility) for each survey respondent in the NHWS as a function of four types of variables: (1) indicator variables for the five conditions of interest, (2) measures of comorbidity, (3) age and gender, and (4) sources of unmet need. The coefficients on the medical condition variables are measures of the disutilities of those conditions, after controlling for age, gender, and comorbidities. The sources of unmet need analyzed were financial barriers to care, treatment quality, and treatment adherence. These variables were interacted with the indicator variables for each of the five conditions, which allowed the effect of access to and use of available technologies to vary across conditions. Mathematical details of the regression model are included in the Additional file 1.

### Using the statistical model to predict utility

The statistical model was used to predict utility for categories of YLD. Predictions were made separately for each respondent in the data who reported having one of the five conditions. Utility scores by category are mean predictions by age and gender across respondents. Mean utility was calculated separately for each of the five conditions.

We first estimated *current disease burden* by predicting utility, given variables for each respondent were equal to their observed values. Counterfactual scenarios were used to estimate the remaining three subcategories. In the first two counterfactual scenarios, we used the variables measuring access to and use of care to examine the extent to which current technology reduces unmet need. We estimated the *disease burden untreated* by assuming that each respondent switched from observed care to no care. That is, we predicted utility for each respondent when the values of each of the four access and utilization variables were set to their “worst” values (i.e., low adherence, costs prevent medication use, used cost-cutting strategy, doctor is not attentive to needs and concerns). Conversely, the *achievable disease burden* category was estimated by assuming that each respondent switched from observed care to perfect care by setting the access and utilization variables to their “optimal” values. In the remaining counterfactual scenario, we estimated the *no disease burden* category by assuming that each respondent no longer had the disease in question and that respondents received optimal treatment. The increase in utility from the *current met need* category to the *no disease burden* category is consequently equal to the estimated disutility of the disease for patients using current treatments optimally.

### Estimating years of quality-adjusted life lost to disutility (YLD)

YLD by age, gender, and unmet need category were evaluated for each of the five diseases using Eq. 2 by multiplying disutility weights for a given category by the prevalence of disease in that category. Total YLD was calculated by summing across age and gender.

## Results

Table 1 provides information related to the impact of the variables used in the statistical model on utility and highlights the frequency of particular demographic characteristics, comorbidities, medical conditions, and poor access and use of care. The results show that the access and utilization variables have the expected association with utility. Utility was lower for respondents who reported that costs created barriers to medication use and higher for respondents with better adherence to their medications or who believed that their physicians were attentive to their needs and concerns. The relationship between disease severity and utility is also of note. Respondents who did not use medications had lower utility scores, suggesting that not using medication is a proxy for disease severity. Moreover, utility was negatively correlated with the number of comorbidities.

**Table 1:** Utility scores of 2013 NHWS survey respondents by demographics, disease severity, access and utilization

	EU5			US		
	N	%	Mean utility	N	%	Mean utility
Age group						
18–34	16,964	27%	0.73	19,421	26%	0.73
35–54	24,005	39%	0.73	26,293	35%	0.75
55–74	19,266	31%	0.72	25,821	34%	0.76
75+	1765	3%	0.69	3465	5%	0.75
Gender						
Female	33,305	54%	0.71	38,711	52%	0.74
Male	28,695	46%	0.74	36,289	48%	0.75
Number of comorbidities						
0	26,067	42%	0.77	26,363	35%	0.79
1	13,836	22%	0.73	14,800	20%	0.76
2	9056	15%	0.70	10,915	15%	0.75
3 or more	13,041	21%	0.64	22,922	31%	0.69
Cost prevented medication use						
No	54,733	88%	0.73	58,012	77%	0.77
Yes	7267	12%	0.65	16,988	23%	0.68
Used a cost cutting strategy for medications						
No	47,138	76%	0.74	44,343	59%	0.78
Yes	14,862	24%	0.66	30,657	41%	0.71
Doctor was attentive to needs and concerns						
No	8565	14%	0.70	6717	9%	0.71
Yes	53,435	86%	0.73	68,283	91%	0.75
Adherence						
*Breast cancer*						
Not using medications	487	64%	0.67	740	72%	0.73
Low/medium adherence	166	22%	0.61	148	14%	0.67
High adherence	104	14%	0.67	145	14%	0.78
*COPD*						
Not using medications	291	30%	0.64	399	20%	0.65
Low/medium adherence	423	43%	0.58	892	45%	0.60
High adherence	268	27%	0.62	712	36%	0.66
*Hepatitis C*						
Not using medications	356	85%	0.65	598	91%	0.66
Low/medium adherence	50	12%	0.58	62	9%	0.56
High adherence	13	3%	0.62	15	2%	0.68
*Parkinson’s*						
Not using medications	36	33%	0.63	35	24%	0.63
Low/medium adherence	49	45%	0.57	71	48%	0.58
High adherence	25	23%	0.63	42	28%	0.71
*Rheumatoid arthritis*						
Not using medications	215	31%	0.63	251	21%	0.64
Low/medium adherence	313	45%	0.58	581	48%	0.60
High adherence	165	24%	0.57	369	31%	0.64

**Fig. 2:**
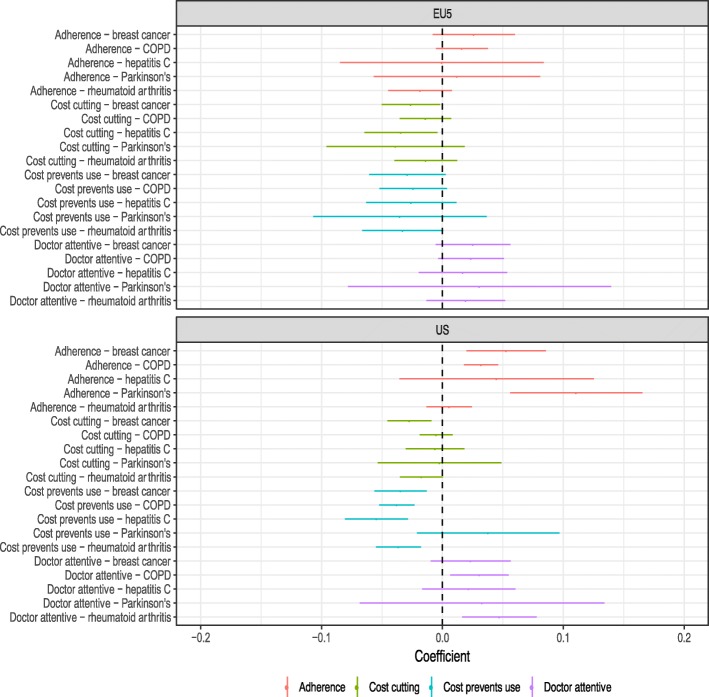
Association between access, utilization variables and utility. Notes: Dependent variable is each respondent’s utility score. Error bars are 95% confidence intervals

The impact of the access and utilization variables on utility is shown in Fig. 2. The figure reports regression coefficients from the full statistical model, which controls for age, gender, and disease severity. We would have expected the coefficients on the two cost-related variables to be negative, the coefficient on the adherence variables to be positive, and the coefficient on the physician attentiveness variable to be positive. This was generally observed, with a few exceptions, such as the coefficient on high adherence for respondents with RA in the EU5. Estimates of all regression coefficients in the model are reported in the Additional file 1: Table S1.

**Fig. 3:**
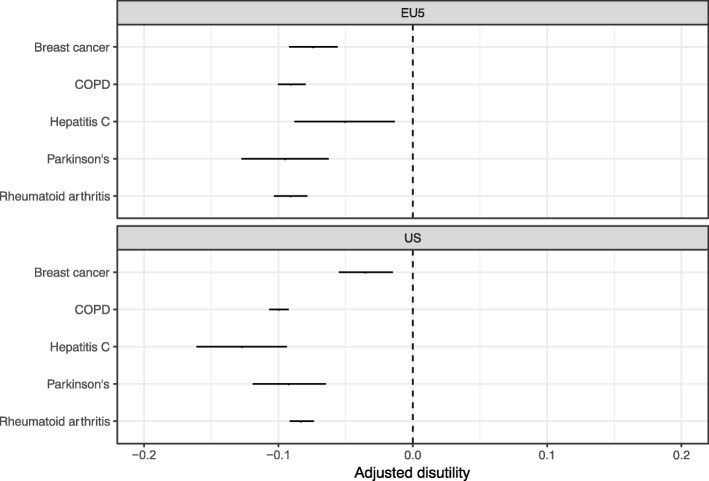
Adjusted disutility of disease. Notes: Adjusted disutility in the figure is predicted disutility from the model averaged across all individuals in the NHWS, with the access and utilization variables set to their observed values. Error bars are 95% confidence intervals. Standard errors were calculated by using the simulation-based method described in Mandel et al. [30] In particular, coefficients from the regression model were simulated 1000 times using an asymptotic normal distribution. For each of the 1000 simulations, disutility was predicted for all survey respondents with a given medical condition. Mean disutility was then calculated across individuals for each draw and the mean, 2.5% quartile, and 97.5% quartile of mean disutility were used to generate the point estimates and 95% confidence intervals in the plot

Figure 3 examines the impact of the five medical conditions on utility. The estimates are measures of the disutility of each condition given current access to and use of technology, since they were calculated with all variables in the statistical model set to their observed values. The coefficient on each condition was negative in both the EU5 and the US, implying that the conditions decreased utility, ceteris paribus. Overall, disutilities ranged from around − 0.03 to − 0.13 and were similar in both geographic regions. The coefficients were estimated precisely, as the 95% confidence intervals did not cross zero. The disutility estimates can be interpreted as the amount that patient utility would increase if a condition were completely eliminated.

**Fig. 4:**
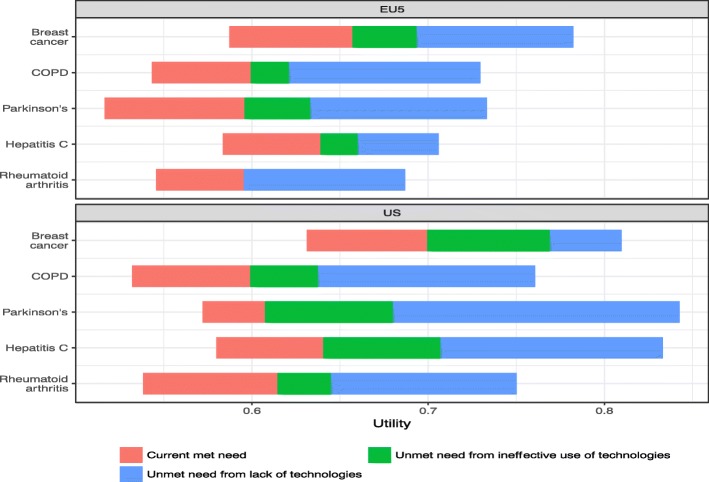
Predicted utility by categories of met and unmet need. Notes: This figure shows predicted utility by subcategory of met and unmet need by disease and geographic area. Total need is measured by utility and is broken into current met need, unmet need from ineffective use of technologies and unmet need from a lack of technologies

We predicted utility using our statistical model by subcategory of met and unmet need in Fig. 4. For each of the diseases, the figure decomposes total need as measured by utility (i.e., ignoring prevalence) in the EU5 and US into the proportion that is currently being met, could be met given current technologies, and could be met given future technologies. In most cases, current technologies increased utility considerably, although significant unmet need remains. While ensuring better access to and use of available technologies could reduce some of the unmet need gap, this gap, with the exception of breast cancer in the US, was driven mostly by lack of technologies.

**Fig. 5:**
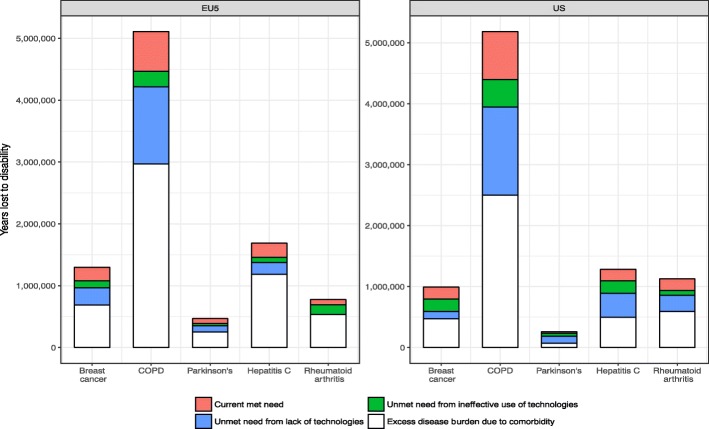
Years lost to disutility by categories of met and unmet need. Notes: This figure displays estimates of YLD, which combines the utility data (as shown in Fig. 4) with prevalence data from the GBD for each disease and geographic area. Utility is broken into current met need, unmet need from ineffective use of technologies, unmet need from a lack of technologies, and extent to which unmet need would still exist because of comorbidities, even if the condition in question was completely eliminated

Figure 5 displays estimates of YLD, which combines the utility data shown in Fig. 4 with prevalence data from the GBD. The figure contains the same three subcategories of met and unmet need as in Fig. 4, but adds a fourth category, which is the extent to which unmet need would still exist because of comorbidities, even if the condition in question was completely eliminated. Although the extent of unmet need caused by comorbidities is substantial, future innovations and more effective use of current technologies could substantially reduce disease burden. The disease burden for COPD is worth highlighting given its large magnitude; for example, our results imply that more efficient use of current technologies in the US would reduce YLD by 451,447 and that future technologies could reduce YLD by another 1,446,507.

## Discussion

Our study presents a new conceptual framework for understanding unmet need and demonstrates how it can be applied empirically to compare the magnitude of unmet need across conditions and countries. The proposed approach presents considerable technical challenges, largely due to data requirements, but these can be overcome using comprehensive international datasets such as the NHWS. The results presented have good face validity. For example, it is reassuring that the amount of unmet need associated with financial barriers to care was higher in the US, which has multiple public and private payers, than in European countries with universal health coverage.

More comprehensive research efforts in this area could demonstrate where societies and private companies would reap the greatest benefits from improving access to currently available drugs and technologies, improving the application of such therapies, and investing in the development of new therapies. While such efforts could ultimately help prioritize resource allocation by disease and type of intervention, efforts are still needed to overcome some of the limitations of this study. Below, we outline these limitations and highlight areas where future research would be most beneficial.

### Data limitations

As with many modeling efforts, many of the limitations of our work concern data scope and quality. For instance, since the NHWS is a self-reported survey, the manner of recruitment and survey administration may represent a form of selection bias, and the responses may be subject to recall bias or misinterpretation of the questions. Furthermore, while the NHWS uses the same survey in multiple geographical locations, it is not undertaken everywhere. There are also sample size limitations, as some rare conditions in smaller countries do not provide enough data to effectively generate usable results. We chose two large regions – the US and Europe – because they represent a large proportion of global medical technology utilization. We concentrated on a selection of non-communicable diseases that impose a large morbidity burden in order to simplify the analyses and demonstrate the value of using HR-QOL disutility as our main outcome. It is inevitable that including mortality in the analyses would have changed the relative size of unmet need for different conditions, for example, increasing the relative size of unmet need in breast cancer.

The NHWS is cross-sectional, which limits the causal interpretation of our findings. Specifically, we assumed that the access, utilization, and disease variables have a causal effect on utility after controlling for age, gender, and comorbidities. To the extent that this is not the case, our counterfactual scenarios may be biased. New longitudinal datasets with multiple observations per individual would be welcome and allow future researchers to leverage time-series variation to estimate model parameters.

Our model focused on three drivers of achievable unmet need: financial barriers, patient-perceived quality, and adherence. There are other drivers that may be relevant, which we could not address with the NHWS data. These include geographical access barriers, regulatory barriers such as unapproved technologies, and medical errors unknown to the patient. Although these factors will likely be correlated with the variables we currently measure, the likely effect of including these in any future models will be to increase the level of achievable unmet need and shrink the level of unmet need caused by lack of technology.

Finally, our study measured the drivers of achievable unmet need directly from patients. This has some limitations because patients are unaware of many aspects of unmet need, particularly around the quality of their treatment. There is also the danger of ‘common method variance’ where associations between patient-reported health status and the sources of unmet need are driven by underlying response tendencies.

### Model limitations

It should be noted that this model does not put a value on unmet need. In a standard economics framework, the aggregate willingness to pay (WTP) of individuals for a technology that eliminates a given disease reflects the value of that technology to society. In recent years, economics has made considerable progress in incorporating societal distributive concerns [24, 25]. To the extent that the measurement of unmet need is important, insofar as it helps society prioritize healthcare resources, measuring societal value is an approach to measuring unmet need grounded in economic theory. Economists have long argued that the value individuals place on the alleviation of disease relies heavily on context, including the relative severity of disease, patient age and gender, and proximity to end of life, among other factors [26, 27]. The value that society places on the alleviation of disease can be combined with the QALY estimates of disease burden from our model to provide a richer measure of unmet need, but this is outside the scope of this current model.

Further, the current model cannot be used to prioritize different types of investments. For instance, our results do not allow decision-makers to determine whether they should invest in policies that improve access to care or that increase adherence. Likewise, our current model does not inform decisions about whether to invest in more effective use of current technologies or disease prevention. The latter may particularly relevant for diseases like COPD that are often caused by risky health behaviors such as smoking. Future research that considers both new drivers of achievable met need and that quantifies their relative importance would be valuable.

### Strengths and applications

The most insightful and interesting results from our model relate to differences between the US and Europe in terms of the underlying levels of disutility experienced across diseases and also the degree to which different causes of unmet need proliferate across territories and diseases.

A surprising finding from our model was that it predicted that baseline utility levels across all diseases if treatment was optimally effective – i.e. all disease burden of the disease in question was addressed – were higher in the US than in the EU5, as can be seen in Fig. 4. There are a number of possible explanations for these differences including that there is less comorbidity in the US population (which appears untrue given the results presented in Table 1) or that there are cultural or regional differences in how people value or experience HR-QOL in the two geographic regions (which seems likely given that utility scores are higher in the US even conditional on the number of comorbidities). Estimates of met need for four diseases: COPD, Parkinson’s, RA and hepatitis C were all very similar when comparing the EU5 and US. The only met need scale that differed significantly was in breast cancer, where numerous studies have highlighted that both earlier detection and overtreatment is common in the US compared with the EU5 [28, 29]. Unsurprisingly, the size of ‘unmet need due to lack of access to technologies’ was larger in the US than in the EU5 countries, as all five of these countries have a healthcare system based around universal access to healthcare, whereas the US system, at least in the years studied, has a significant proportion of its population suffering from a lack of access to healthcare.

In addition, Fig. 5 highlights the overall scale of the different diseases in each territory. In both panels, COPD dwarfs the other diseases. Clearly this was a function of the prevalence of COPD more widely, but it is clear that COPD should be a priority for the development of new health technologies. Interestingly, the disease with the second highest unmet need from lack of technologies was hepatitis C, but this is due to the fact that the year of data collected was prior to the introduction of a series of highly-efficacious antiretrovirals. Similarly, perhaps reflecting the timing of the study, the bulk of unmet need in RA was lack of access to current technologies in the US, whereas it was a lack of effective technologies in the EU. This could be attributable to the wider range of RA drugs available in the US in 2013 than in almost all of the EU5 countries.

These results only highlight a small set of select diseases, but they do have potential to draw considerable insight into the often-neglected aspect of the informational requirements of resource allocation and priority setting in healthcare, not just for present healthcare policy, but also for directing investment into future technologies in different disease areas, in terms of potential return on investment.

## Conclusion

An accurate assessment of the health needs of a population is critical to allocating scarce healthcare resources across diseases. Unmet need has emerged as a concept to define how the health of a population differs from what is achievable. Although an intuitively obvious need, a conceptual framework for both defining and measuring unmet need has remained lacking. Unmet need may take a short-term perspective that reflects the gap between current disease burden and disease burden that would exist if all individuals had access to available treatment, or it may take a long-term perspective that reflects the gap between current disease burden and normal aging. Such a perspective is important to identifying areas where research and development into new technologies may be needed most.

Our approach attempts to quantify these categories in two different regions across a select set of diseases in terms of HR-QOL. While the approach is not without limitations, and there is no guarantee that additional investments will lead to effective new treatments, we believe our framework and model can inform health systems in terms of directing investment activity between improving access to medicines or increasing investment in research and development across and within disease areas. Future research should refine our methodology, expand our analyses to other disease areas, and consider the use and collection of new data.

## Additional file

Additional file 1: Technical Appendix. (DOCX 38 kb)

## Abbreviations

COPD: Chronic Obstructive Pulmonary Disease
DALY: Disability-Adjusted Life Year
EU5: United Kingdom, Germany, France, Italy, and Spain
GBD: Global Burden of Disease Study
HR-QOL: Health Related Quality of Life
HRS: Health and Retirement Study
MEPS: Medical Expenditure Panel Survey
MMAS-8: Morisky Medication Adherence Scale, 8-Item
NHWS: National Health and Wellness Survey
QALY: Quality-Adjusted Life Year
SF-36v2: Short-Form 36-item (version 2)
SF-6D: Short-Form Six-Dimension
US: United States
WTP: Willingness to Pay
YLD: Years Lost to Disutility
