# Supplementary material for: An empirical tool for estimating the share of unmet need due to healthcare inefficiencies, suboptimal access, and lack of effective technologies
Source: BMC Health Serv Res. 2019 Feb 11;19:113. doi: 10.1186/s12913-019-3914-7 (PMC6371562; doi:10.1186/s12913-019-3914-7)
Supplement: Supplementary file 1 — Technical Appendix. (DOCX 38 kb) [file 12913_2019_3914_MOESM1_ESM.docx]

Additional file 1

**Calculation of utility scores**

Surveyed individuals answered the Short-Form 36-item (version 2; SF-36v2) health survey, a standardized questionnaire used to assess patient health status. The SF-36v2 was then converted into a reduced version known as the Short-Form Six-Dimension (SF-6D) consisting of six multi-level dimensions. An SF-6D health state is defined by scores on each of the six dimensions, ranging from optimal health (111111) to a state worse than death known as the “pits” (6456555). In total, there are 18,000 possible health states defined by the SF-6D.

The SF-6D health states were converted into preference-based scores using the algorithm from Brazier et al.[1] Specifically, Brazier et al. had previously valued 249 of the possible health states with a sample of 611 members of the UK population using standard gamble and estimated an econometric model capable of predicting health states for all 18,000 states based on this sample. We used the Brazier et al. econometric model to predict utility scores for each respondent in the NHWS.

**Utility regressions**

Each individual’s expected health utility score is modeled as

$$E\left[ utility \right]=\alpha_{0}+\sum_{i=1}^{4} \beta_{i}age^{i}+\gamma female+\sum_{i=0}^{3} \delta_{i}comorbdities_{i}+\sum_{i=1}^{5} {\kappa_{i}m}_{i}+\sum_{i=1}^{5} \lambda_{i}norx_{i}+\sum_{i=1}^{5} \sum_{j=1}^{4} \mu_{j}z_{j}m_{i}$$

where

- $\alpha_{0}$, $\beta_{1},\ldots,\beta_{4}$, $\gamma$,$\delta_{0},\ldots,\delta_{3}$,$\kappa_{1},\ldots,\kappa_{5}$, $\lambda_{1},\ldots,\lambda_{5}$, and $\mu_{1},\ldots,\mu_{4}$ are regression coefficients;
- $age$ is single-year of age; $female$ is an indicator variable equal to 1 if a respondent is female and 0 if male; $comorbidities_{0},\ldots,comorbidities_{3}$ are indicator variables for the number of comorbidities (0, 1, 2, and 3+);
- $m_{1},\ldots,m_{5}$ are indicator variables for each of the five diseases of interest;
- Each $norx_{i}$ is an indicator variable equal to 1 if a respondent reporting having condition $i$ but did not taking any medications for it and $0$ otherwise;
- $z_{1},\ldots,z_{4}$ are the four access and utilization variables (high adherence, costs prevented a respondent from taking medications, respondent using a cost-cutting strategy for their medications, doctor is attentive to needs and concerns).

**Table S1. Coefficients from the OLS multivariate regression in the 2013 NHWS**

|  | **EU5** | |  | **US** | |
| --- | --- | --- | --- | --- | --- |
|  | **Estimate** | **Standard error** |  | **Estimate** | **Standard error** |
| **Variable** |  |  |  |  |  |
| Intercept | 0.4557 | 0.0314 |  | 0.4107 | 0.0267 |
| Age | 0.0290 | 0.0029 |  | 0.0310 | 0.0025 |
| Age^2^ | -0.0010 | 0.0001 |  | -0.0010 | 0.0001 |
| Age^3^ | 0.0000 | 0.0000 |  | 0.0000 | 0.0000 |
| Age^4^ | 0.0000 | 0.0000 |  | 0.0000 | 0.0000 |
| Female | -0.0241 | 0.0011 |  | -0.0035 | 0.0011 |
|  |  |  |  |  |  |
|  |  |  |  |  |  |
| *Number of comorbidities (ref = 0)* |  |  |  |  |  |
| 1 | -0.0475 | 0.0015 |  | -0.0520 | 0.0015 |
| 2 | -0.0814 | 0.0017 |  | -0.0848 | 0.0017 |
| 3 or more | -0.1384 | 0.0016 |  | -0.1536 | 0.0015 |
|  |  |  |  |  |  |
| *Not using medication for condition* |  |  |  |  |  |
| Rheumatoid arthritis | 0.0691 | 0.0129 |  | 0.0333 | 0.0111 |
| Breast cancer | 0.0590 | 0.0125 |  | 0.0281 | 0.0129 |
| Parkinson’s | 0.1024 | 0.0306 |  | 0.0789 | 0.0292 |
| Hepatitis C | 0.0051 | 0.0213 |  | 0.0651 | 0.0189 |
| COPD | 0.0457 | 0.0105 |  | 0.0421 | 0.0085 |
|  |  |  |  |  |  |
| *High adherence to medication (ref = low/medium)* |  |  |  |  |  |
| Rheumatoid arthritis | -0.0184 | 0.0135 |  | 0.0056 | 0.0096 |
| Breast cancer | 0.0260 | 0.0174 |  | 0.0527 | 0.0168 |
| Parkinson’s | 0.0120 | 0.0352 |  | 0.1107 | 0.0279 |
| Hepatitis C | -0.0006 | 0.0430 |  | 0.0448 | 0.0411 |
| COPD | 0.0161 | 0.0109 |  | 0.0319 | 0.0072 |
|  |  |  |  |  |  |
| *Cost prevented medication use* |  |  |  |  |  |
| Rheumatoid arthritis | -0.0329 | 0.0171 |  | -0.0364 | 0.0095 |
| Breast cancer | -0.0290 | 0.0161 |  | -0.0347 | 0.0110 |
| Parkinson’s | -0.0353 | 0.0366 |  | 0.0378 | 0.0300 |
| Hepatitis C | -0.0258 | 0.0190 |  | -0.0546 | 0.0133 |
| COPD | -0.0242 | 0.0143 |  | -0.0378 | 0.0074 |
|  |  |  |  |  |  |
| *Used a cost-cutting strategy* |  |  |  |  |  |
| Rheumatoid arthritis | -0.0137 | 0.0133 |  | -0.0173 | 0.0091 |
| Breast cancer | -0.0260 | 0.0124 |  | -0.0273 | 0.0093 |
| Parkinson’s | -0.0388 | 0.0292 |  | -0.0023 | 0.0261 |
| Hepatitis C | -0.0344 | 0.0154 |  | -0.0061 | 0.0124 |
| COPD | -0.0140 | 0.0108 |  | -0.0052 | 0.0070 |
|  |  |  |  |  |  |
| *Doctor attentive to needs and concerns* |  |  |  |  |  |
| Rheumatoid arthritis | 0.0193 | 0.0166 |  | 0.0472 | 0.0157 |
| Breast cancer | 0.0253 | 0.0158 |  | 0.0233 | 0.0169 |
| Parkinson’s | 0.0306 | 0.0555 |  | 0.0328 | 0.0516 |
| Hepatitis C | 0.0169 | 0.0187 |  | 0.0218 | 0.0197 |
| COPD | 0.0236 | 0.0139 |  | 0.0306 | 0.0124 |

**References**

1. Brazier, J., J. Roberts, and M. Deverill, *The estimation of a preference-based measure of health from the SF-36.* J Health Econ, 2002. **21**(2): p. 271-92.
